# Genetic Modulation Therapy Through Stem Cell Transplantation for Human Immunodeficiency Virus 1 Infection

**DOI:** 10.7759/cureus.1093

**Published:** 2017-03-13

**Authors:** Varshil Mehta, Divya Chandramohan, Shivika Agarwal

**Affiliations:** 1 Department of Cardiology, Mount Sinai Hospital, New York, USA; 2 Department of Internal Medicine, SRM Medical College, Kancheepuram, Tamil Nadu, India; 3 Department of Forensic Medicine, ESIC Medical College, Faridabad, India

**Keywords:** hiv treatment, ccr5 resistance, genetic modulation, hematopoietic stem cell transplantation

## Abstract

Highly active anti-retroviral treatment has changed the dimensions of the outcomes for patients suffering from human immunodeficiency virus (HIV)/acquired immunodeficiency syndrome (AIDS). However, HIV infection is still an ailment which is spreading throughout the world extensively. Given the confinements of the present restorative methodologies and the non-availability of any strategic vaccination against HIV, there is a squeezing need to build a therapeutic treatment.

Viral tropism for HIV includes CD4+ cells, macrophages, and microglial cells, and it is through binding with co-receptors C-C chemokine receptor type 5 (CCR5) and C-X-C chemokine receptor type 4 (CXCR4). While these cell types are present in all individuals, there are rare cases that stayed uninfected even after getting exposed to an overwhelming load of HIV. Research revealed a homozygous 32-base pair deletion (Δ32/Δ32) in CCR5. After careful consideration, a hypothesis was proposed a few years back that a cure for HIV disease is possible, through hematopoietic stem cells transplantation from a donor homozygous for the CCR5-Δ32 deletion.

Hematopoietic stem cell (HSC) based quality treatment may serve as a promising tool as these perpetual, self-renewing progenitor cells could be modified to oppose HIV infection. If done properly, the changed HSCs would offer the permanent creation of genetically modified cells that are resistant to HIV infection and/or have improved hostility to viral action which will eventually clear the contaminated cells.

The purpose of this review is to concentrate on two facets of HSC genetic treatment for potentially life-threatening HIV infection: building HIV-resistant cells and designing cells that can target HIV disease. These two strategic approaches can be the frontline of a quality treatment plan against HIV infection and, as an individual treatment or a combination thereof, has been proposed to possibly destroy HIV altogether.

## Introduction and background

As indicated by the Joint United Nations Programme on Human Immunodeficiency Virus/Acquired Immune Deficiency Syndrome (UNAIDS), there were roughly 36.7 million individuals living with HIV/AIDS towards the end of 2015. Of these, 1.8 million were children (<15 years old). An alarming 2.1 million people were recently contaminated with HIV in 2015. This encompassed 150,000 <15-year-olds. The greater part of these children live in sub-Saharan Africa and were contaminated by their HIV-positive mothers amidst pregnancy, labor, or breastfeeding [1]. Although there has been an increase in awareness regarding HIV infection, people remain uninformed about the same in many parts of the world, especially in the slums of developing countries [2-3].

Despite the success of HIV therapy through the combined antiretroviral treatment delivered, HIV infection continues to be a resilient ailment which keeps spreading rapidly around the world. Highly active antiretroviral treatment (HARRT) has been proven to be of maximum benefit for those individuals who have not yet succumbed to an advanced stage of disease, provided it is started earlier in the disease process and is adhered to properly. In such cases, there is a notable decrease in the plasma HIV viral load to low or even imperceptible levels in many patients. This has changed what generally was conceived as a lethal ailment to a constant infirmity.

Notwithstanding this achievement, antiretroviral treatment is not yet completely impeccable. Chronic inflammation and immune dysfunction frequently result post-usage, and subsequent emerging proof demonstrates that there is enigmatic viral replication in scattered lymphoid organs while the therapy is ongoing [4]. Alongside the other harmful effects of antiretroviral medications, this adds to the increasing detriment of non-AIDS morbidity and mortality [5-6].

The HAART regimen requires strict adherence to therapy and numerous patients are non-compliant. Additionally, in asset-restricted nations, access to treatment is capricious and seldom uninterrupted. Given the shortcomings of the present treatment protocol and the non-availability of a vaccination against HIV, it is imperative to build a therapeutic regimen with a steady rate of success.

A large portion of the general population is susceptible to HIV infection. However, uncommon cases have been noted who stayed uninfected even after getting exposed to an overwhelming load of HIV. Studying these cases have since revealed a homozygous 32-bp deletion (Δ32/Δ32) found in C-C chemokine receptor type 5 (CCR5) [7]. While the CD4 T-cell function remains intact, the deletion rendered cells resistant to HIV attachment, since the ordinarily transmitted R5 viruses utilize the CCR5 receptor together with CD4 to gain entry into cells. This awareness prompted the advancement and utilization of a new class of entry inhibitors that bar CCR5 collaboration with the HIV envelope; this a proposed plausible cure of HIV disease, accomplished through an allogeneic stem cell transplantation from a donor homozygous for the CCR5-Δ32 deletion [8].

Consequently, hematopoietic stem cell (HSC) based quality treatments have developed as a promising tool, as these cells could be modified and tuned to oppose HIV infection [9-10]. If they are effectively engrafted, the changed HSCs could offer consistent, long-haul creation of genetically modified cells that are resistant to HIV infection and/or have improved hostility to viral action to clear contaminated cells. If the host is successfully repopulated with an HIV-resistant hematopoietic framework to not provide attachment sites even given a heavy viral load, then a lasting cure can be accomplished.

This review concentrates on the factors of HSC genetic treatments which potentially help in treating/preventing life-threatening HIV infection by building HIV-resistant cells and making cells which could target the virus. These two approaches could replace the frontline treatment against HIV infection and can possibly help in eliminating HIV altogether.

## Review

### Co-receptors for the passage of HIV into CD4+

**Figure 1:**
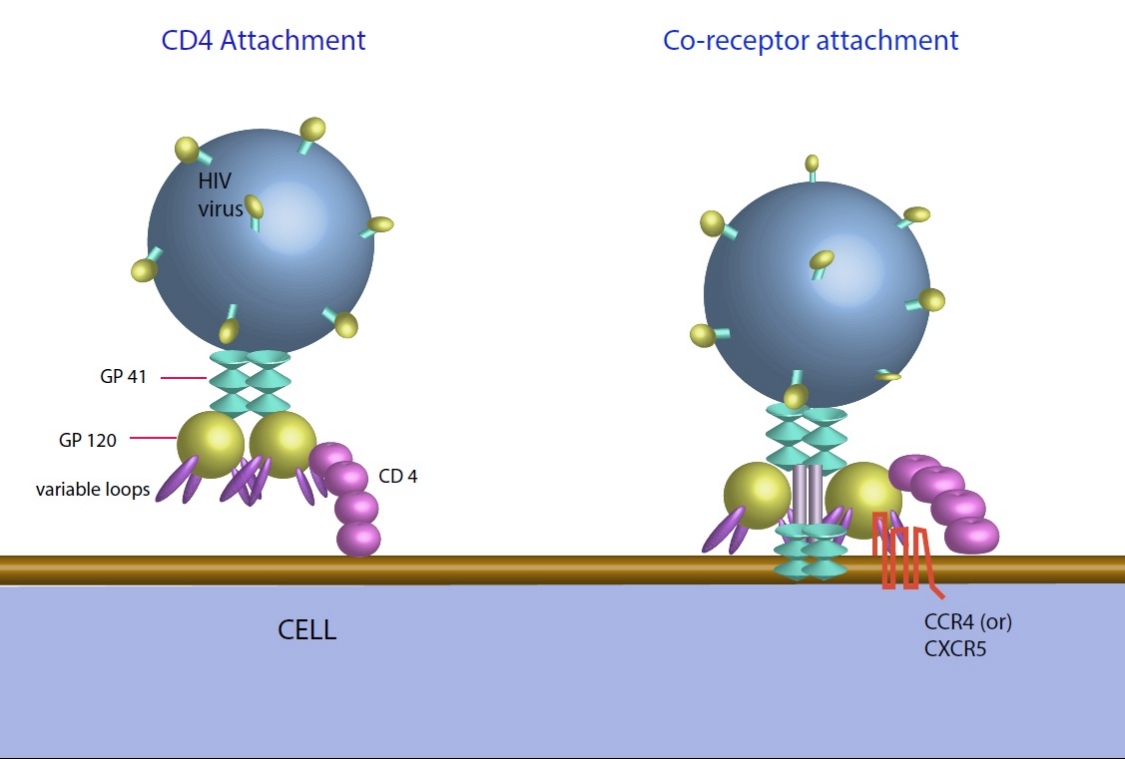
HIV attachment process Adapted from US National Institutes of Health - National Institute of Allergy and Infectious Diseases [14].

Viral replication requires cellular gene expression processes and activated CD4+ cells which are the primary targets of replicative HIV infection. Thus, HIV disease leads to a decrease in activated memory CD4+ T-cells, the greater part of which exist in the gastrointestinal (GI) mucosa [11-13]. The HIV viral access into CD4+ cells requires proper interaction with a cell surface co-receptor - for the most part, either CCR5 or CXCR4 [8]. The picture is depicted below in Figure 1 [14].

### CCR5 co-receptor

Out of the two, CCR5 is more basic as a co-receptor, and CCR5 tropism represents a larger share of transmittable HIV-1 strains [15]. The chemokine CCR5 is a vital component in the immune system, where it is profoundly expressed on macrophages and CD4 T-cells [16]. The chromosome 3 in the p21.3–p24 zone expresses the gene for CCR5. In the coding area of CCR5, there are known to be no less than 23 generally uncommon alleles [17]. As expressed, it has been ascertained that people who are homozygous for a deletion of 32 base sets of the CCR5 gene (Δ32) are to a great extent resistant to HIV infection [7]. Once HIV-infected, people with a solitary duplicate of the Δ32 mutation additionally have slower illness progression [18]. Homozygosity of CCR5-Δ32 in Caucasians was found to be around 1%, with heterozygosity being anything up to 20% [19]. The recognizable proof of these subjects has made CCR5 an alluring option against antiviral remedial measures. For instance, the Maraviroc, a negative allosteric modulator of the CCR5 receptor, was developed, and it emerged as a fruitful entry inhibitor [20]. Be that as it may, antiviral resistance can still evolve with this medication, and a more lasting procedure focusing on CCR5 would be a more compelling methodology.

### CXCR4 co-receptor

C-X-C chemokine receptor type 4 (CXCR4) are associated with various physiological processes, for example, B-cell and T-cell development and also as part of cardiovascular and cerebral systems. However, due to its wide expression in numerous cell types, it is not a plausible HSC gene-based remedial target [21].

In a study by Allers, et al., it was emphasized that CXCR4 surface expression on peripheral and mucosal CD4+ T-cells is not disabled in the CCR5Δ32/Δ32 stem cell transplant (SCT) patient. They additionally discovered that CD4+ cells, once altered at a genetic level, had a half-life of about a year and terminated at a slower rate in contrast with the genetically unmodified CD4+ cells when the ART treatment ceased [8].

### Engineering stem cell transplantation

Through Host Cellular Genes Modulation

**Figure 2:**
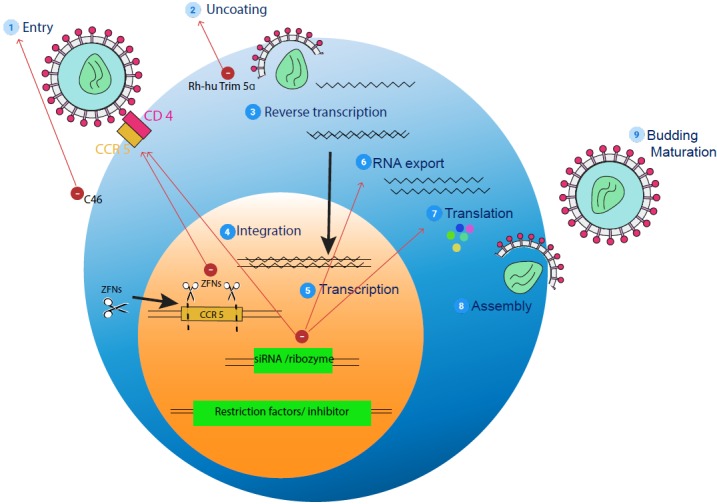
HIV life cycle and strategies to engineer HIV-resistant cells (1) Entry: HIV enters the host cell by attaching envelope glycoproteins, gp 120 and gp 41 to CD4, utilizing CCR5 or CXCR4 as a co-receptor. Fusion into the cell membrane is inhibited by the membrane-bound form of the T20 inhibitor, the peptide C46. Small interfering RNAs or siRNAs and zinc finger nucleases (ZFNs) can be designed to inhibit entry. (2) Uncoating: HIV particle uncoats and releases its RNA into the cell. Rh-hu Trim 5 alpha can help by inhibiting uncoating. (3) Subsequently, the RNA undergoes reverse transcription. (4) It now gets incorporated into the genome by  integration. (5) The RNA is  transcribed and exported out of the nucleus (6). (7) It is further translated into protein, which occurs in the cytoplasm of the cell. Short interfering RNA (siRNA) can inhibit RNA export and translation. (8) The translated viral particle undergoes assembly and leaves the cell to transfect multiple more cells. Adapted from Zhen A and Kitchen S [22].

As part of strategical approaches to hinder the process of HIV replication at different levels, HSCs are transformed to make them HIV-resistant (Figure 2) [22]. These methods can be categorized into three main strategies: the foremost earmarks the cellular genes required for viral replication, and the focal point of this article will be the newest advances targeting the CCR5 co-receptor which is a requisite for viral entry; the second one targets the HIV gene expression itself; and the last one, using the restriction factors of the host and fusion inhibitors, introduces genes that interfere with HIV replication.

The phenomenal case of the “Berlin Patient”, the first patient who was declared cured of an HIV infection, further emphasized the prospects of targeting CCR5 as a potential treatment for HIV infection. This patient had received a bone marrow transplant for acute myeloid leukemia from a donor homozygous for CCR5Δ32/Δ32. [8, 23]. HIV was undetectable in the patient for years even consequent to discontinuation of combination antiretroviral therapy. This was attributed to the engrafted donor cells that are believed to have imparted long-term control of the HIV infection post-transplantation.

Despite the success in the aforementioned case, this approach is unsuitable as a mainstay treatment strategy. The rare odds of finding a human leukocyte antigen-matched, CCR5Δ32/Δ32 homozygous donor (especially within the non-European populations), and the substantial morbidity and mortality risk attributable to allogeneic stem cell transplantation, have significantly restrained this strategy, to be used only as a last resort for patients with AIDS-associated malignancies.

Gene therapies that modify autologous peripheral blood T-cells or HSCs can be used to mimic the CCR5Δ32/Δ32 phenotype seen in the Berlin Patient. In particular, genetically modified HSCs can be used to engineer HIV-1 resistant immunity that would prevent ongoing viral infection. To that end, several gene therapy approaches are being tested to reduce CCR5 expression or disrupt the CCR5 gene. These include using ribonucleic acid interference (RNAi) [24], using ribozymes to reduce CCR5 RNA levels [25], or using intrabodies [26] and intrakines [27] to target the CCR5 protein directly. RNA interference can be achieved through the stable expression of CCR5 short hairpin RNA (shRNA) from lentiviruses.

A recent study by Shimizu, et al. demonstrated that the transduction of human CD34+ hematopoietic cells with a CCR5-specific shRNA expressing lentivirus appeared to have no major adverse effect on T-cell development in humanized bone marrow/liver/thymus (BLT) mice. Down regulation of CCR5 was found in both human T-cells and monocytes/macrophages in systemic lymphoid tissues, and CCR5 tropic HIV-1 replication was effectively inhibited ex vivo [28].

To directly impair the genomic sequence of CCR5 and subsequently block its expression is another possible approach. To dismiss CCR5 in autologous CD4 T cells, Bobis-Wozowicz et al., in their study, used zinc-finger nuclease (ZFN) with the help of an adenoviral vector. These ZFNs are actually artificial restriction enzymes that can be programmed to cleave deoxyribonucleic acid (DNA) at specific sites. Since these breaks naturally induce mutations during their repair, the disruption of the ability of targeted allele, in this case CCR5, to make a functional protein, becomes more plausible. Importantly, only transient ZFN expression is required to permanently disrupt the target genes, and thus it confers very little risk of immune elimination because of antigen presentation [29].

About 50% of CCR5 alleles in a pool of primary T-cells were identified as being permanently disrupted by transient ZFN expression in the initial studies done by Perez, et al. [30]. One subsequent study concluded that the CCR5 gene was disrupted in 17% of human CD34+ hematopoietic cells by ZFN via nucleofection using the humanized mouse model, and the modified cells were successfully engrafted in non-obese diabetic (NOD)/severe combined immunodeficiency (SCID)/IL2rγnull (NSG) mice. Successful resistance to R5-tropic HIV infection was conferred to cells with this modification. Evidently, rapid selection for CCR5−/− cells during HIV infection occurred in the mice transplanted with ZFN modified cells, and these mice had significantly lower HIV-1 levels [31]. 

These positive outcomes encouraged several clinical trials of ZFN targeting CCR5 in peripheral T-cells (clinicaltrials.gov: NCR00842634, NCT01044654). According to a recent study, ZFN nuclease when delivered by a recombinant adenoviral vector effectively disrupted >25% CCR5 gene in protein kinase C (PKC) activator pretreated HSCs isolated from granulocyte colony-stimulating factor (CSF)-mobilized adults, and these cells underwent multilineage differentiation both in vitro and in vivo [32].

Through HIV Gene Modulation

HIV genes that are essential for viral replication can also serve as targets for HIV gene therapy. Among them, tat and rev are both transactivators and are critical for HIV-1 infectivity. In stem cells, HIV tat and its overlapping genes have been targeted using hammerhead ribozymes, which are small catalytic RNA molecules engineered to target specific RNA species. A Phase 2 gene therapy trial showed that patients who received tat-vpr-specific ribozyme modified autologous CD34+ cells have higher CD4+ T-cell counts compared to the placebo group, but they have no statistically significant differences in viral load [33].

RNA decoys have also been used to inhibit tat recognition of HIV trans-activating response region (TAR) and inhibit viral replication in vitro [34]. HIV rev, the viral regulatory protein that allows the nucleus to do cytoplasmic transport of unspliced viral RNA, can also be targeted in gene therapy. Dominant mutants or trans-dominant forms of rev have been used to inhibit HIV replication in gene-modified cells [5-6, 34-36]. Decoy rev responsive elements (RREs) have also been tested as potential therapeutic targets and were shown to sequester rev in a clinical trial [9-10, 37].

Based on the pattern of HIV gene expression, different stages of the viral replication cycle can also be targeted by RNA interference (RNAi). All viral transcripts, including those encoding tat, rev, gag, pol, nef, vif, env, vpr, as well as long terminal repeats (LTR), are susceptible to RNAi down regulation in cell lines [15, 38]. A substantial problem of clinical application of RNAi is the virus’s high mutation rate and its ability to escape targeted therapy [7,39]. Therefore, as described previously, targeting cellular factors such as CCR5 by RNAi may be a more attractive gene therapy strategy than targeting viral transcripts.

Through Use of Modified T-Cell Cloned Receptor (TCR)

Apart from designing HIV-resistant cells, immunity too plays a pivotal role in containing HIV infection. One of the strategies is to genetically modify peripheral blood cells with a molecularly cloned T-cell receptor (TCR) or a chimeric molecule to boost the host's antiviral immunity that can redirect the cells to aim at HIV antigens.

The peripheral cells of the patient can be modified by peptide-specific TCR from cloned reactive T-cells [8, 40-42]. In one study, a TCR from a patient that had a sustained and robust CTL response against HIV gag SL9 peptide was molecularly cloned. When genetically modified, CD8 cells were introduced to the primary CD8 cells via transfection; they showed enhanced and polyfunctional immune responses against the viral antigen with an increased capacity to control HIV infection [42].

Numerous hurdles that were encountered in previous studies and trials can be circumvented by stem cell-based anti-HIV immune therapy. The stem cell-based “redirection” approach also has some advantages over previous strategies. In this approach, proper thymic selection of the modified cells and exclusion of endogenous TCR surface expression will be allowed by molecularly cloned TCR, eliminating the chances of generating self-reactive TCR through mispairing [43].

Also, this therapy would allow a long-lived and renewable immunity system to continuously produce antiviral cells that could ultimately lead to HIV eradication. Recently, a study concluded that viral replication was significantly lowered after humanized mice were transplanted with human HSCs genetically modified with an anti-HIV SL9 TCR in comparison with control mice after HIV exposure [44].

The genetic modification of HSCs with a TCR entitle the cells to differentiate in vivo and the stem cells modified by TCR differentiate in to mature CD8+ cells in various tissues [44]. This study highlighted the potential capability of redirecting anti-HIV immunity using HSC recent advances in identifying antigen-specific TCR shall make it possible to molecularly clone multiple TCR from each patient quickly [45].

The cloned TCR, which matches the patient’s human leukocyte antigen (HLA) type and viral genome, could be used to genetically modify patient’s HSCs which can subsequently allow the development of evolved and sustained modified CD8+ T-cells capable of targeting and eradicating HIV-infected cells.

### Potential risks and adverse effects

It has already been observed that CCR5 Δ32 mutation protects against HIV, but may also play a negative role in post-infection inflammatory processes, which can not only injure tissue but can also create further pathology [46]. Homozygous patients for CCR5 Δ32 were found to be at higher risk for a neuroinvasive form of tick-borne encephalitis [47]. In addition, functional CCR5 shall be required to prevent symptoms after infection with West Nile virus (WNV). CCR5 Δ32 was also associated with the development of early symptoms and more prominent clinical manifestations after infection with WNV [48]. In a study done in mice, the viral load in the central nervous system and mortality increased after an infection with WNV [49]. However, with further enhancement of technology related to the modulation of stem cells, it is expected that the adverse effects and potential risk will diminish [50].

## Conclusions

With the latest technology available, the gene-modifying system has been improved significantly and has now become an appealing strategy for HIV treatment. Of late, HSC-based anti-HIV gene therapy seems promising. This treatment can definitely give long-lasting and renewable immune cells that are either HIV resistant or insusceptible to HIV entry. With further research and with more funds for carrying out this research, HSC-based gene-modification therapy can assuredly acquire great results and, may be, can also lead to a cure followed by the eradication of HIV.
